# Generation of vector vortex wave modes in cylindrical waveguides

**DOI:** 10.1038/s41598-023-37890-8

**Published:** 2023-07-08

**Authors:** Md Khadimul Islam, Pawan Gaire, Arjuna Madanayake, Shubhendu Bhardwaj

**Affiliations:** 1 Department of Electrical and Computer Engineering at Florida International University, Miami, FL 33174 USA; 2 Department of Electrical and Computer Engineering at University of Nebraska-Lincoln, Lincoln, NE 68588 USA

**Keywords:** Engineering, Electrical and electronic engineering

## Abstract

In this paper, we propose a method to generate Vector Vortex Modes (VVM) inside a metallic cylindrical waveguide at microwave frequencies and demonstrate the experimental validation of the concept. Vector vortex modes of EM waves can carry both spin and orbital angular momentum as they propagate within a tubular medium. The existence of such waves in tubular media can be beneficial to wireless communication in such structures. These waves can carry different orbital angular momentum and spin angular momentum, and therefore, they feature the ability to carry multiple orthogonal modes at the same frequency due to spatial structure of the phase and polarization. In essence, high data rate channels can be developed using such waves. In free space, Orbital Angular Momentum carrying vortex waves have beam divergence issues and a central field-minima, which makes these waves unfavorable for free space communication. But vector vortex mode waves in guided structures do not suffer from these drawbacks. This prospect of enhancement of communication spectrum in waveguides provides the background for the study of vortex wave in circular waveguides. In this work, new feed structures and a radial array of monopoles are designed to generate the VVM carrying waves inside the waveguide. The experimental findings on the distribution of the amplitude and phase of the electromagnetic fields inside the waveguide are presented and the relationship between the waveguide fundamental modes and VVMs are discussed for the first time. The paper also presents methods for varying the cutoff frequency of the VVMs by introducing dielectric materials in the waveguide.

## Introduction

Unlike conventional waves (such as linearly, circularly, and elliptically polarized waves) which possess spatial homogeneous field distribution, the Vector Vortex Waves (VVMs) have inhomogeneous field distribution and exhibit cylindrically symmetric polarization state and/or spiral phase front. These specific phase and polarization qualities allow generation and transmission of simultaneous orthogonal modes in a media, which is useful for channel multiplexing for high data-rate wireless communication. The phase singularity of such waves is due to twisting phase front represented by $e^{\pm jm\phi }$, where *m* is topological charge and $\phi$ is the azimuthal angle around the axis of propagation^1–6^. On the other hand, polarization singularity can be expressed by defining the polarization using a vector $\alpha (\phi )=l\phi +\alpha _0$, where *l* is the polarization order and $\alpha _0$ is the initial polarization. For an OAM carrying vortex wave, $m=l\sigma$ where $\sigma =+1$ for left-handed circular spin and $\sigma =-1$ for right-handed circular spin. Through the use of these properties, VVMs can offer theoretically infinite number of eigen states and therefore have capability of providing enhanced spectral efficiency in wireless communication links. The properties of these waves are of scientific and engineering interests due to their applications in communication^7–10^, quantum technology^11–13^ and radar and imaging^14–17^, which have arisen from similar applications in the optical frequency band. VVMs offer better SNR for free space communication in comparison to conventional waves^18^. Additionally, those modes can enable terabit links mitigating the turbulence issues in optical frequencies^19–21^.

Inspired from the success of vortex waves at optical wavelengths, vortex waves have also been investigated for their applications in free-space communication^22–33^. So far their applications have been limited due to the beam diffraction, resulting in the well-known broadside-amplitude-NULL issue^34^. In essence, this requires large receiver antennas with low aperture efficiency. This issue has limited the applications of vortex waves in the microwave and millimeter wave (MMW) frequency bands. State of the art research has been focused on generating a limited number of vortex modes in free-space^35–41^. At MMW frequency bands, the antenna’s physical sizes being smaller, there is more interest in communication systems^42–44^. Specifically, mode-generation^23,45^, multiplexing^46,47^ is experimentally demonstrated and using these, communication links with data-rates up to 32 Gbps^48–50^ have been shown.

In this paper, we propose methods of generation of vector vortex wave modes in tubular and cylindrical structures suitable for applications in microwave frequency range. In many RF applications, waveguides are often used to feed the main radiating unit^51–53^, hence generation of vector vortex modes in waveguides simplifies the design of antennas for generating such modes. For example, a horn antenna connected to a cylindrical waveguide carrying vector vortex modes, allows radiation of VVMs in free space without use complex antenna or metasurfaces. Ability to radiate multiple vortex modes through a same feeding structure such that multiplexing occurs in the feeding structure is a desirable technology which simplifies the design complexity of antenna structures used for generating multimodal vortex waves. Similarly, cylindrically excited modes can be used in conjugation with a reflectarray or a reflector antenna to generate free-space VVMs. Another potential application of the presented work is towards the wireless communication in tubular structures. In future, such modes in cylindrical waveguides could be used in transmission of wireless signal in closed tubular structures as well. Currently mining tunnels, subways, and other innovative transport concepts such as Hyperloop rely on Fresnel Zone wireless links. Fresnel Zone links use spherical wavefront inside tubular structures and therefore are bandwidth and distance limited. This limitation arises from the interference from multiple reflections from the enclosing walls^54,55^ which can be overcome by employing waveguide modes, and be spectrally enhanced by employing VVMs.

Vector vortex modes are characterized by an electric or magnetic field vector that is rotating around an axis, while the wave also exhibits a spiral azimuthal variation of phase. The spiral behavior can be generated by manipulating the phase and polarization of the wave. In prior works, we observe use of metamaterials, phase plates, printed lenses, and use of specific orientation of antennas^56–59^ to achieve generation of such waves. Within cylindrical waveguides, prior works have shown the theoretical origin of such waves by combining fundamental modes^52,60^. Preliminary results that illustrate the numerical simulation of such scenarios are shown in^17,51^, however, a systematic method to generate such modes in practice has not been shown in prior works.

We show that through careful selection of a radially aligned antenna and appropriate phase feeding, such modes can be generated in cylindrical hollow metallic waveguides. We study this requirement of antenna elements and phase in this work. The numerical and experimental results are shown to demonstrate such waves for the first time. In this work, we also show manipulation of a mode’s cut-off frequency, by using dielectric filling in the partial section of the waveguides. The paper is organized as follows. We first highlight the advantages of the vector vortex modes. Then the theoretical origin of the modes, which is established to be from a combination of fundamental TE modes are discussed. A method to create vector vortex modes inside hollow metallic circular waveguide is developed through observation of electric and magnetic fields afterwards. Then the experimental results are presented, and conclusions and future works are discussed in the last section.

## Relevance of vortex modes for tubular communication

In this paper, we show that the Vortex wave modes allow multiplexing of many orthogonal modes using the same antenna structure. Such a system provides physical layer security for wireless links due to multiplexing of orthogonal phase fronts, and energy efficiency arising from a smaller loss when compared to Fresnel zone communication. Although multi-modal communication in tubular structure is also possible through TE/TM modes which are orthogonal by definition, many vortex modes are generated through same antenna array and we propose a systematic method for generation and detection of such vortex modes. Therefore this form of multimodal communication offers simplicity and ease of implementation. Furthermore, identification of methods of generation of such waves in waveguides is beneficial for the research towards free-space angular momentum waves, since the fields in a waveguide can be radiated using a horn antenna or fed to a reflector antenna. Such systems could allow integrated multiplexing and radiation of the multiple vortex modes through a common antenna, greatly simplifying multiplexing and transmission of OAM modes.

**Figure 1:**
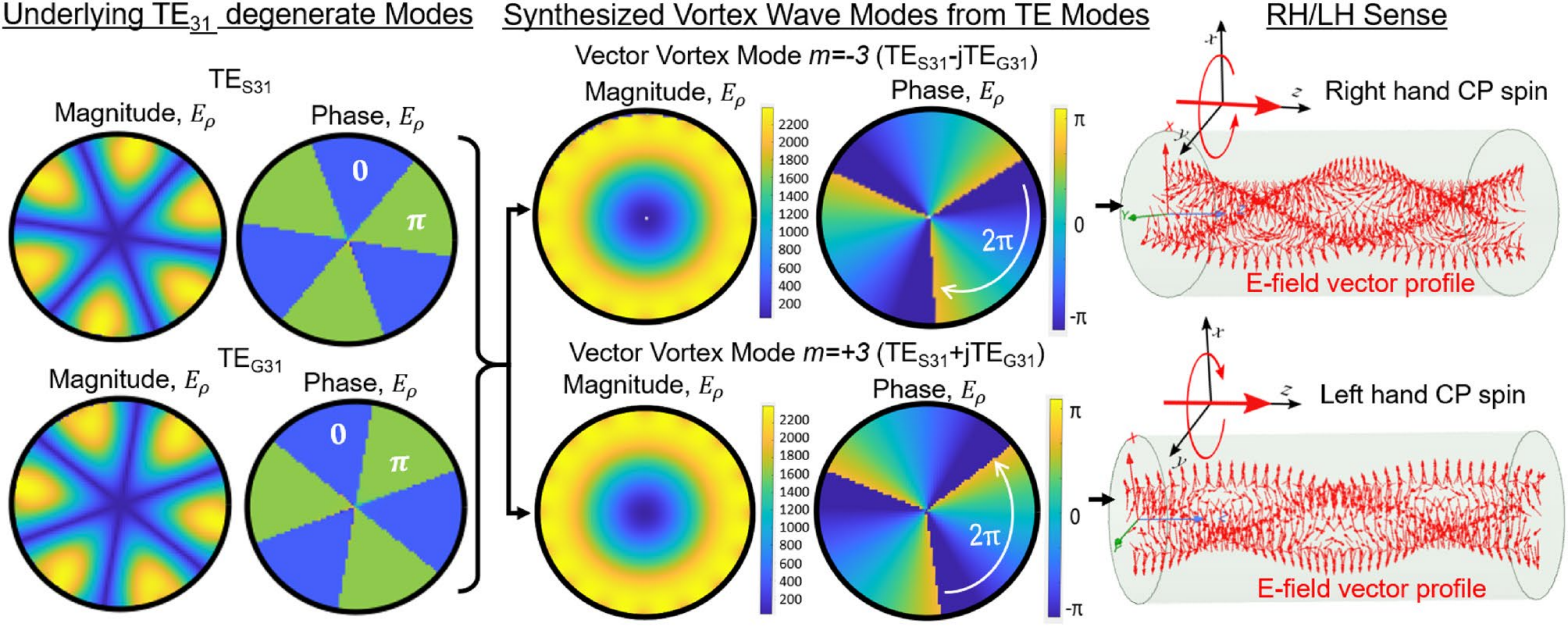
Numerical simulation of synthesis of vector vortex modes from degenerate TE modes in a perfectly electrical conductor (PEC) cylindrical waveguide. Vector vortex modes were generated by exciting the two TE modes with $90^o$ phase difference between the modes. Radius of the waveguide = 20 mm. Frequency = 12 GHz.

## Theoretical origin

### Angular momentum of propagating electromagnetic (EM) field

An EM field may have angular momenta in several forms^61^ and for the cylindrical waveguide, these may take the forms of orbital angular momentum $(M_{OAM})$, spin angular momentum $(M_{SM})$ and surface angular momentum $(M_{Surf})$^60^. The volume density (or length density) of sum of these momenta provide the total angular momenta of the waves, expressed as

1 $$\begin{aligned} M_{AM}=M_{OAM}+M_{SAM}+M_{Surf}. \end{aligned}$$

Surface angular momentum of modes in waveguides is zero when integrated across the cross-section^60^, and therefore cylindrical modes are associated with only a combination of spin and angular momentum^60^ shown as

2 $$\begin{aligned} M_{AM}=M_{OAM}+M_{SAM}. \end{aligned}$$

The orbital angular momentum of a propagating field is related to the spiral wavefront present in the electromagnetic wave, while spin angular momentum of the wave is associated to circular polarization (CP) of the wave.

### Vector vortex modes versus orbital angular momentum modes in waveguides

Plane and spherical waves used in free-space communication may have a spiral wavefront and such waves are commonly known as vortex waves or orbital angular momentum waves. Such waves may or may not have a spin angular momentum, in addition to the orbital angular momentum. However, in hollow waveguides, orbital angular momentum and spin angular momentum are coupled and therefore must simultaneously exist^60^. That is, a propagating wave inside a hollow waveguide must have spiral phase wavefront $(M_{OAM}\not =0)$ and circular polarization ( $M_{SAM}\not =0$), simultaneously. This justifies the use of the term *vector* vortex wave, since *vector* refers to a non-homogeneous polarization of the circularly polarized wave in addition to the vorticity of the phase. This observation is important in developing the proposed antenna structure needed for generating and receiving the vortex waves inside tubular structures.

### Modal synthesis of vector vortex mode waves

In a significant development^60^, it was shown that the vector vortex modes inside metallic circular waveguides are indeed a superimposition of fundamental transverse electric (TE) or transverse magnetic (TM) modes. Since this analysis is provided in the prior work, we only summarize the findings and then propose a method for generation of such waves. We consider TE modes to demonstrate the vector vortex waves, but similar considerations can be made for TM modes by using a suitable magnetic analogue of an electric radiator, e.g. use of slot antenna instead of dipole antenna.

Considering the radial component of electric field $E_\rho$ to represent the wave mode, the two degenerate modes of the TE mode, namely $TE_{Smn}$ and $TE_{Gmn}$, are solutions to Maxwell’s equations^62^, and are written as

3 $$\begin{aligned} E_{\rho mnS}= & {} \frac{jk_o^2n}{k_{mn}^2\rho }J_m(k_{mn}\rho )cos(m\phi )e^{-jk_zz} \end{aligned}$$

4 $$\begin{aligned} E_{\rho mnG}= & {} \frac{jk_o^2n}{k_{mn}^2\rho }J_m(k_{mn}\rho )sin(m\phi )e^{-jk_zz} \end{aligned}$$

Here $k_o$ is the free space wave number, $J_m (x)$ is the *m*th order Bessel function of first type, $\rho$ is the radial distance from the center of the waveguide, $k_{mn}n=p_{mn}^{'}/a$ is the propagation constant along the radial direction in the cross-section of radius *a*, for the *mn*th mode. $p_{mn}^{'}$ is the *n*th solution of the equation $J_m^{'}(x)=0$, where prime ($'$) represents the derivative with respect to the argument. $k_z$ is the propagation constant along the direction of propagation, given by $k_z=\sqrt{(k_o^2-k_{mn}^2 )}$. An example of $\hbox {TE}_{31}$ the two degenerate modes is shown in Fig. 1 left.

**Figure 2:**
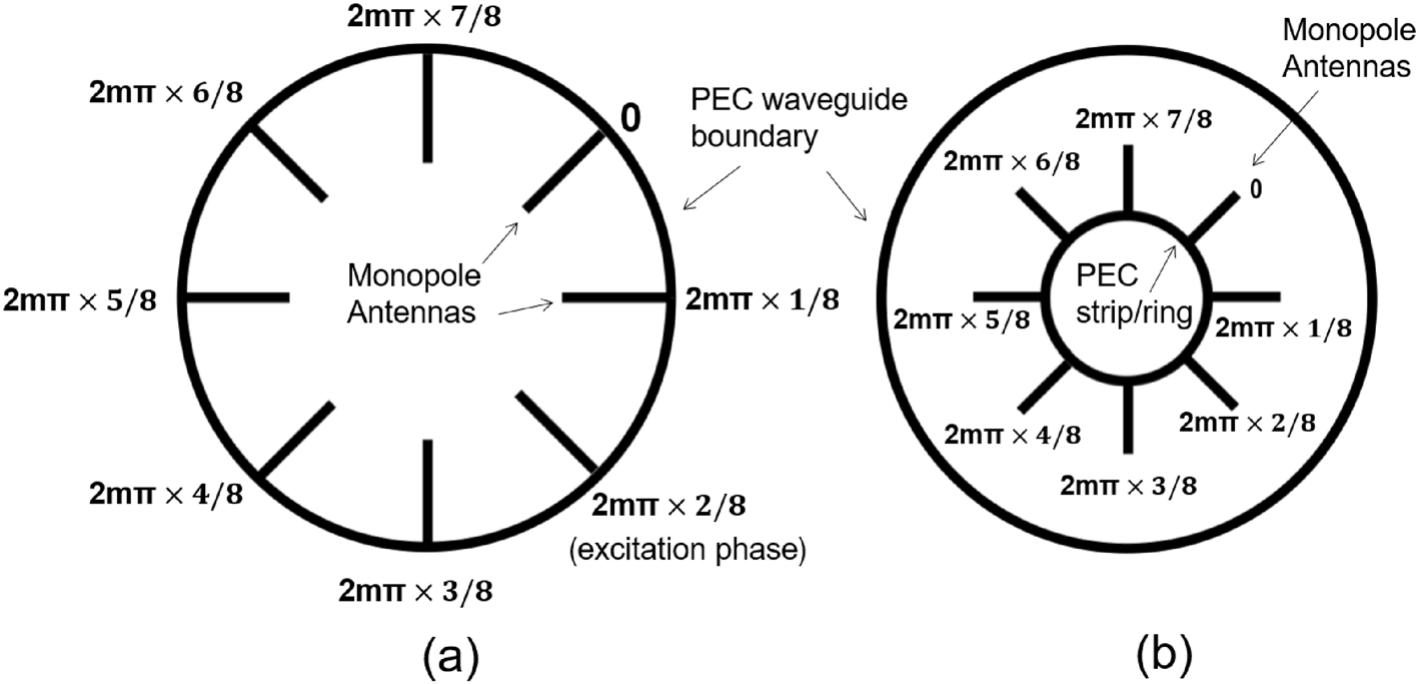
Two potential methods for exciting vector vortex modes within circular waveguides. The first configuration is preferable due to ease of practical implementation.

Vector vortex modes arise by superposition of the two degenerate TE modes, where a phase lag of $\pm 90^o$ is introduced between them as shown in Fig. 1. The radial field component of the superimposed E-field is expressed as

5 $$\begin{aligned} \begin{aligned} E_{\rho m} ={}&\frac{jk_o^2n}{k_{mn}^2\rho }J_m(k_{mn}\rho )\{cos(m\phi )\pm jsin(m\phi )\}e^{-jk_zz} \\ =&\frac{jk_o^2n}{k_{mn}^2\rho }J_m(k_{mn}\rho )e^{\pm jm\phi }e^{-jk_zz}. \end{aligned} \end{aligned}$$

Vector vortex mode’s vorticity is apparent in the azimuthal phase variation with a coefficient *m*. At the same time, we observe that the radial direction of the fields represents a non-homogeneous polarization, i.e. polarization is not linear, leading to the name *vector* vortex wave mode.

**Figure 3:**
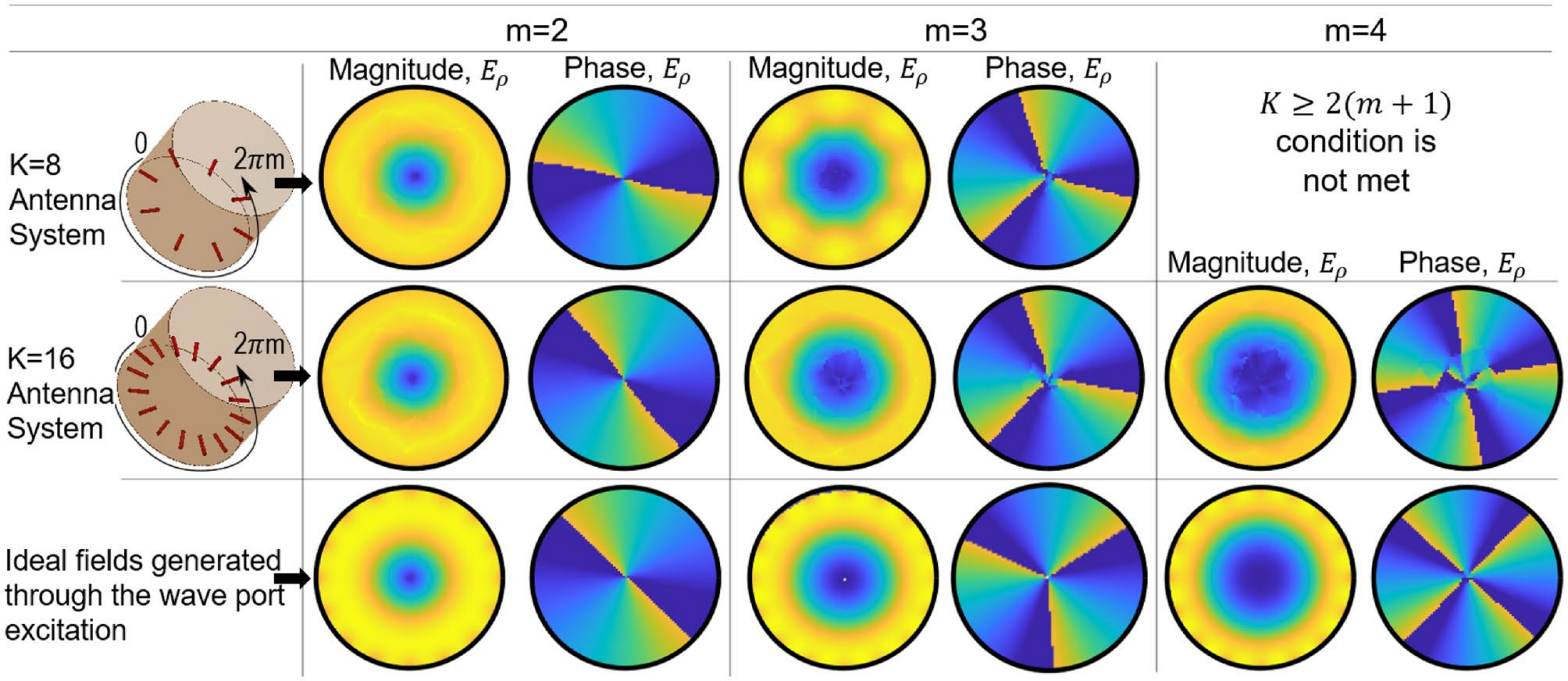
Verification of the electric-field component($E_{\rho }$) and its phase generated from the antenna configuration through full-wave simulations for the cases of K = 8 and K = 16. For a mode-generation for mode m, a progressive phase of $2\pi km/K$. is applied at the *k*th element. Parameter choices: radius of waveguide a = 20 mm, Frequency of the plot: m = 2 at 6GHz, m = 3 at 11 GHz, m = 4 at 14 GHz.

### Understanding field profiles

In Fig. 1 the synthesis of a vector vortex mode for $m=\pm 3$ is shown from its degenerate $\hbox {TE}_{31}$ components using numerical finite element simulations with wave-port excitation. Here the degenerate modes are excited using wave-ports with TE modes in Ansys HFSS simulation tool. As noted, although TE modes have radial component of the field, they exhibit minima and maxima along the azimuthal variation due to sinusoidal and cosine variation of the field. On the other hand, due to superposition with a phase lag, $|E_{\rho m}|$ is uniform along azimuthal direction with a spiral phase. A vector representation of the electric field shows that the wave is indeed analogous to RH or LH circularly polarized wave which also refers to the spin angular momentum (Fig. 1, right).

Therefore, through FEM based full wave simulations, we here confirmed the initial analysis shown in^60^, while also confirming that the spiral phase front (non-zero orbital angular momentum) and a circular polarization (non-zero spin angular momentum) are indeed coupled with one another in circular waveguides.

## Synthesis of vector vortex modes in circular cross-sections

### Antenna configuration for generating vortex modes in cylindrical waveguide

Through a visual understanding of field analysis in the cross-section of the circular waveguide, an antenna configuration to generate the vector vortex modes can be derived. To elaborate this, a method for creating the degenerate $\hbox {TE}_{Sm1}$ and $\hbox {TE}_{Gm1}$ modes is first considered. Electric field vectors of this mode will show *m* maximas, *m* minimas and 2*m* field NULLs along the azimuthal direction. Such a field can be excited with a circular array of monopole antennas which are radially located, and that are aligned to coincide with the direction of the field component $E_\rho$ with the maxima and minima field positions. The maximum and minimum values of the field can be obtained by assigning phase values 0 and $\pi$ respectively, to the corresponding antennas. For example, $\hbox {TE}_{S31}$ and $\hbox {TE}_{G31}$ modes have 3 minima and 3 maxima positions (Fig. 1 left) those can be obtained by introducing 6 monopole antennas and alternating phase of 0 and $\pi$.

Next to generate the vector vortex mode of index $\pm m$, we must excite both of the corresponding degenerate $\hbox {TE}_{Sm1}$ and $\hbox {TE}_{Gm1}$ modes such that one degenerate mode is offset in phase by an angle of ${\mp } 90^o$ with respect to other. The 2m monopoles coinciding with *m* minima and *m* maxima positions and an alternating phase, are sufficient to produce either degenerate mode, but not both modes with required phase shift. This can be done by increasing the number of antenna elements by two at the least, and then exciting the elements with a progressive phase shift which varies from 0 to $2m\pi$. Upon doing so, alternating phase shifting required by minima and maxima positions is still satisfied, but intermediate phase positions are excited by additional antennas along the circumference. These additional antennas provide the sense of ‘circular movement’ of phase by adding the original modes with required phase shift. There is no maximum limit on the number of required antenna elements, but minimum is governed by the above discussion which results in total antenna elements to be 2 more than the twice of the mode number ($K\ge 2(m+1)$). The required phase and polarization can be generated through two possible antenna configurations as shown in Fig. 2. From a fabrication point of view, the first configuration shown in Fig. 2a is well suited for our experimental demonstrations of the vector vortex waves.

Assuming we use a total of *K* antenna elements, the phase shift applied at the *k*th antenna element is calculated as

6 $$\begin{aligned} \phi _{prof} = 2\pi m\frac{k}{K}. \end{aligned}$$

In essence, the above solution represents a method of reproducing the ideal E-field distribution for $TE_{cgm}$ and $TE_{sgm}$ modes, using the antennas with proper phase assignment and superimposing them to create the vorticity inside the waveguide.

### Criteria for selection of number of elements (K)

Here we identify the minimum number of antenna elements that are required to obtain a certain vorticity-index *m* in the array cross-section. Based on the discussions shown in the previous section and rigorous full-wave simulation, two criteria have been identified.

1. An even number of antenna elements should be used as the number of minima and maxima are even for a given $\hbox {TE}_{m1}$ mode.
2. The lowest *m* order VVM modes can be generated with *K* antenna elements given $K\ge 2(m+1)$ is followed.

For example, to generate modes up to $m=\pm 3$, an 8-element ($K=8$) array should be used, but this array will not provide modes $m\ge 4$. Likewise, a 16-element ($K=16$) antenna array can generate modes up to $m=\pm 7$. These examples of 8 element and 16 element array were numerically verified in Fig. 3, where $K=8$ antenna array is used to generate modes $m=+2, +3$, and $K=16$ antenna array is used for generation of modes $m=+2$, +3 and +4, out of possible seven modes. In each of the cases, the modes are generated by the same antenna set-up but by applying phase progression for different *m* based on Equation (6). This means that more than one mode can be produced through the same antenna array as long as signals associated to those modes are appropriately multiplexed using a multiplexing network.

In Fig. 3 we compare the resulting cross-sectional electric field profile values generated by the proposed antenna configuration with ideal field profile of the associated VVW modes. As shown, for the chosen number of antenna elements, i.e. *K* = 8 and 16, when appropriate excitation phases are applied for modes *m* = +2, +3 and +4, the resulting field profile generated , agrees with ideally expected field profile. The required vorticity in the phase profile is also observed for each case, justifying the use of monopole array-based antenna.

**Figure 4:**
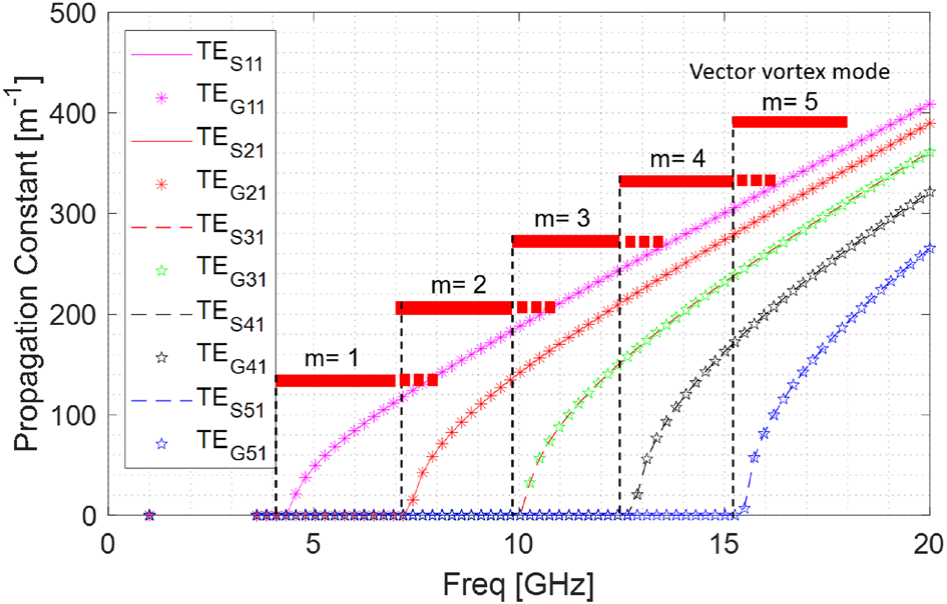
Dispersion diagram showing range of frequencies where proposed antenna scheme can generate the required vector vortex modes with certain vorticity index m.

### Dispersion relation for the modes

An important concern is to identify the cut-off frequency of the vector vortex modes in the circular waveguides. Since vector vortex modes are result of superposition of degenerate TE modes, cut-off frequency and dispersion relation for vector vortex mode with vorticity *m*, is same as the constituent $\hbox {TE}_{m1}$ mode. Figure 4 shows the dispersion relation of the degenerate modes $\hbox {TE}_{S/G(m1)}$ in a metallic cylindrical waveguide.

Note that above dispersion relations are generated through a waveport excitation and represents an ideal dispersion relation given that the applied power is exciting only one of the modes. In practice, when an antenna configurations shown in Fig. 3 is used, the applied power may split between the more than one modes which are possible at that frequency. Therefore, we use the proposed antenna configuration to validate the frequency range within which a specific VVW mode is excited. As shown using a solid bar over frequency ranges, the proposed method is effective in generating modally pure field from the lower bound of the cut-off frequency of the $\hbox {TE}_{m1}$ mode to the upper bound of cut-off of the higher TE mode. Beyond the upper bound of the frequency band (shown with dotted bar), the excited energy may spill over to higher mode and modally pure fields may not be observed. To resolve the spill over issue, a rigorous simulation and experimental study is needed to fully understand the isolation between the modes provided by this antenna configuration and modifying the antenna type or position to produce higher level of isolation between the modes. Additionally, the use of left-hand and right-hand spin will allow further improvement of the isolation. The proposed antenna system can also be used in conjugation with frequency-division multiplexing (FDM) to support traditional methods of spectral enhancements. Antenna matching could be an issue in this scheme while exciting the lower order and higher order modes together at different frequencies. In such case, the currently employed single frequency resonant antennas can be extended to wideband antennas through changes in the shape of monopole or employing impedance matching circuits, however such considerations are not fundamental restriction to the proposed method of generation of the vector vortex modes.

**Figure 5:**
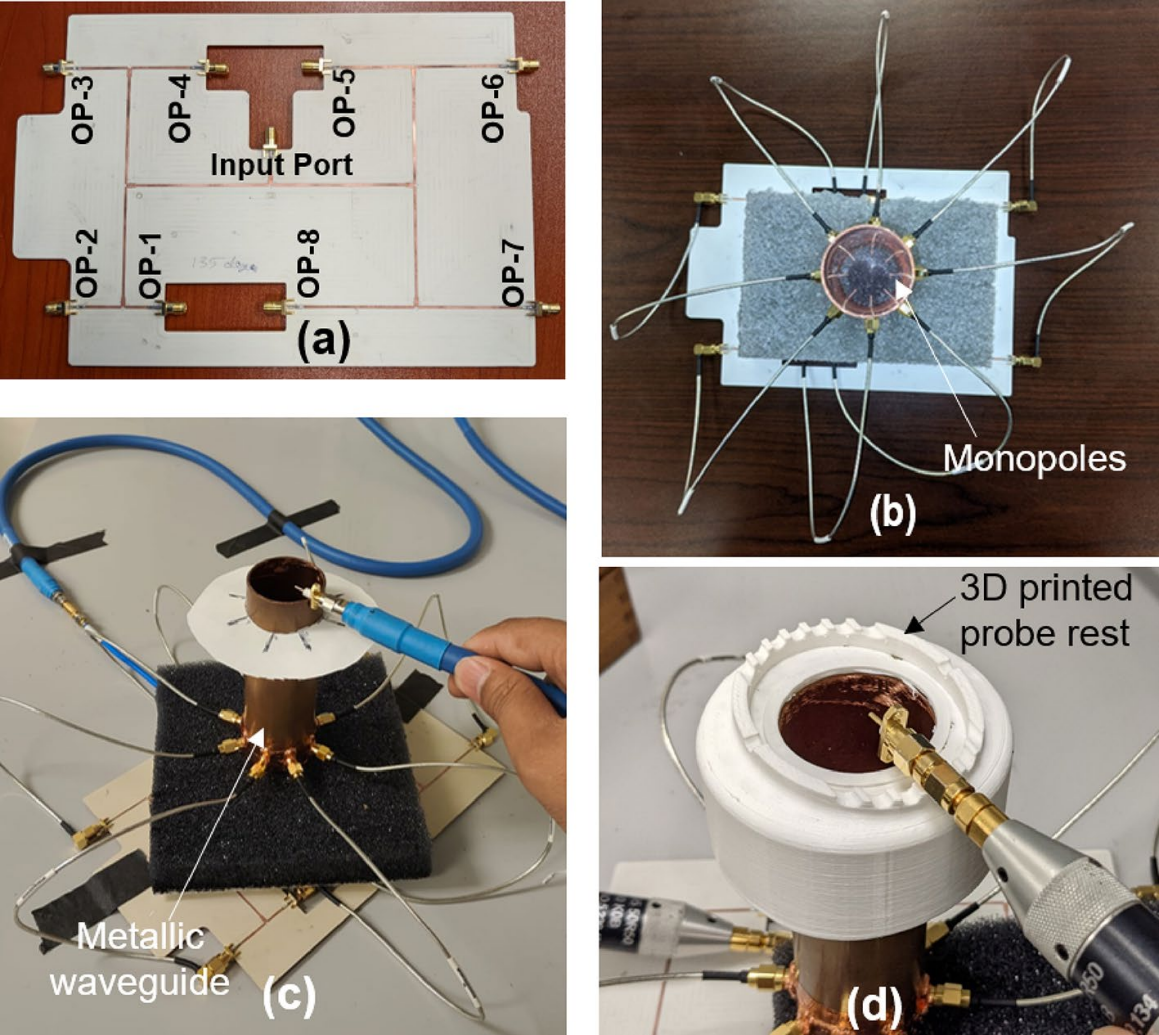
Experimental set-up used for exciting the vector vortex mode with m = 3. (**a**) Roger 4003C printed power divider and phase shifting network to provide 0 to $6\pi$ phase shift (**b**) Phase shifter network connected with the monopoles inside a metallic circular waveguide to provide m = 3 vector vortex mode inside the waveguide, (**c**) Two port network measurement set-up used for measurement of phase and magnitude of the electric field, (**d**) 3D printed probe rest structure to enable the polarization for measurement of $E_x$ and $E_y$ component.

**Figure 6:**
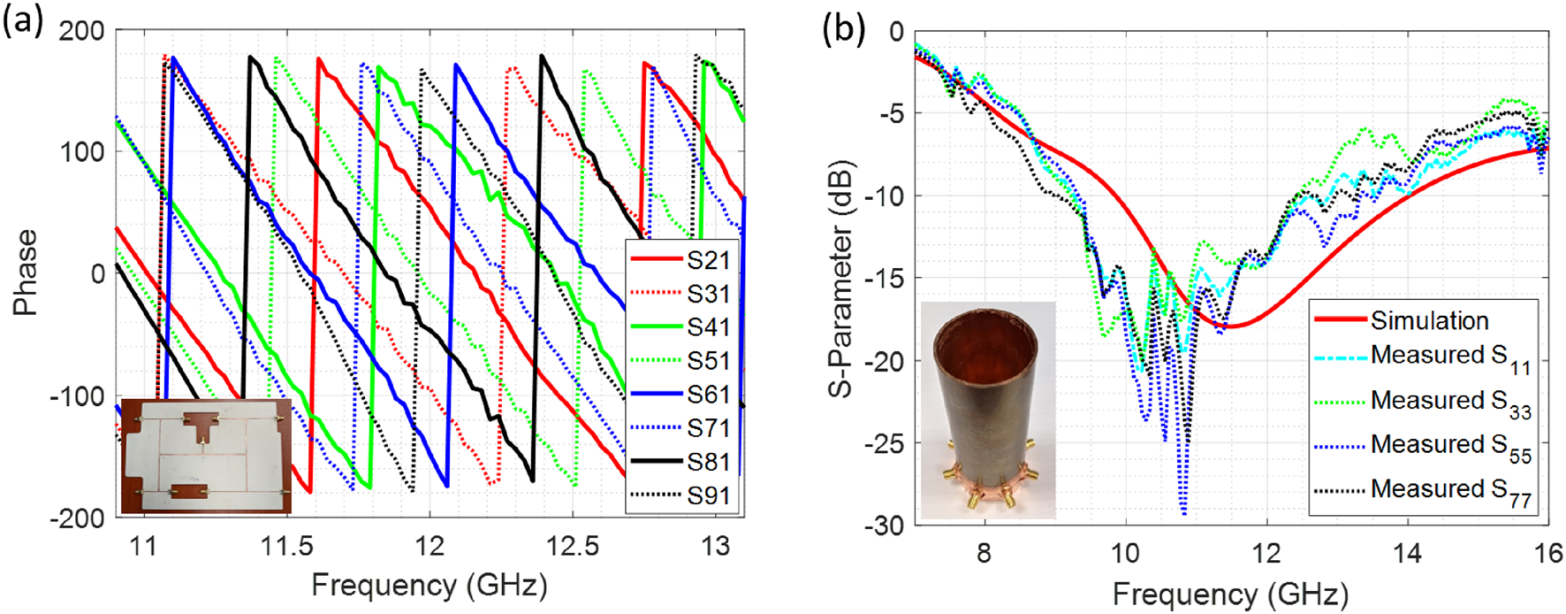
(**a**) Measured phase of the output signal at ports at OP-1 to 8 in (**a**) (labeled as ports 2 to 9 here) and (**b**) comparison of simulated S-Parameter of single unit vs measured S-Parameters of the antennas at the odd positions placed on the periphery of the waveguide.

## Experimental validation

### Measurement of VVM fields in hollow metallic waveguide

To demonstrate the experimental validation of the presented theoretical and simulation work, one to eight power divider, connected with a phase shifting network was designed on a Roger 4003C ($\epsilon _r=3.55$). This microstrip line printed circuit was based on series of T-junctions power dividers, consisting of phase shifters used for true time delay as per selected length of the transmission lines. The photo of the fabricated power divider / phase shifting network is shown in Fig. 5a, and the measured output phase response of the network is shown in Fig. 6a. The measured phase values at different ports showed 2$\%$ to 16$\%$ of phase deviation from the theoretically calculated values at 12 GHz which is within the acceptable tolerance for this experiment. Although, the tolerances may be corrected through optimization and did not impact the field profile significantly for the current demonstrations.

To characterize the impedance matching of the monopole antennas, we measured S-parameter $S_{11}$ these elements. While measuring the response of any of these elements all, the other antenna elements were terminated with 50 $\Omega$ matched loads. Figure 6b shows the measured $S_{11}$ for four of these antenna elements plotted against the simulated value. Due to symmetry of the elements other antenna elements are anticipated to perform similarly. Measurement shows good agreement with the resonance frequency and operation of the monopole radiating over a ground plane is retained, with a small little shift in the resonance frequency observed due the mutual coupling. This shift is acceptable for our demonstrations.

**Figure 7:**
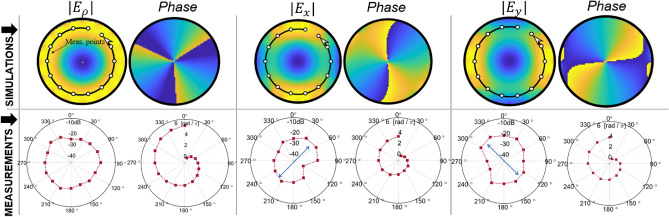
Experimental results validating the excitation of mode m = 3 vector vortex mode within the circular waveguides.

The fabricated power divider-phase shifter circuit is connected to eight monopole antennas radially positioned inside a copper cylindrical waveguide of radius *a* = 20 mm as shown in Fig. 5b. The measurement of the electric field magnitude and phase was conducted at 12 GHz by placing a pin probe in the open section of the waveguide, that is connected to the ports of a vector network analyzer, while the other port provides feeding to the feeding network as shown in Fig. 5c. Thus, this $\hbox {S}_{21}$ measurement provides relative magnitude and phase of the field in the cross-section. The probe is moved in a circular perimeter to complete the measurement of the anticipated spiral phase. For convenience, we conducted measurements along a circle (a linear array of points) which provides us conclusive information on the field’s polarization and phase to determine the existence of the modes within the waveguide cross-section. The locus of measurement points is shown with white points in Fig. 7.

To provide conclusions about the mode present in the waveguide, we have conducted measurements of the $E_x$, $E_y$ and $E_\rho$ components. Measurement of the $E_\rho$ component was conducted by placing the probe normal to the tangent at the circumference of the cross-section. Measurement of electric fields for a specific polarization (such as $E_x$ and $E_y$) was conducted by using a 3D printed structure which would align the probe along *x* or *y* direction as shown in Fig. 5d.

In Fig. 7, the polar plot of the $S_{21}$ magnitude and phase of the fields are shown for the $E_\rho$ component of the field. The phase profile of the field shows a spiral variation (linear variation with azimuthal angle) from 0 to $6\pi$, which verifies the existence of the vector vortex mode $m=-3$. Plotting of the $E_x$ and $E_y$ components of the fields provide an azimuthal variation from 0 to $4\pi$ for mode 3. This is observed in simulation and measurement results as well. This reduced variation of phase to $(m-1)2\pi$ is due to relation between the unit vectors of the polar coordinates and cartesian rectangular coordinates. Since ${\hat{\rho }}={\hat{y}}$ for $y>0$ but ${\hat{\rho }}=-{\hat{y}}$ for $y<0$, and likewise ${\hat{\rho }}={\hat{x}}$ for $x>0$ but ${\hat{\rho }}=-{\hat{x}}$ for $x<0$, there is a $2\pi$ difference of the phase between the $E_\rho$ component and constituent rectangular field components.

We also notice that in the simulations $E_x$ and $E_y$ components have field maxima at the diametrically opposite points within the cross-section. This is also noted in measurements where the polar plots show an oblong circle with major axis aligned towards the direction of the field component being measured.

**Figure 8:**
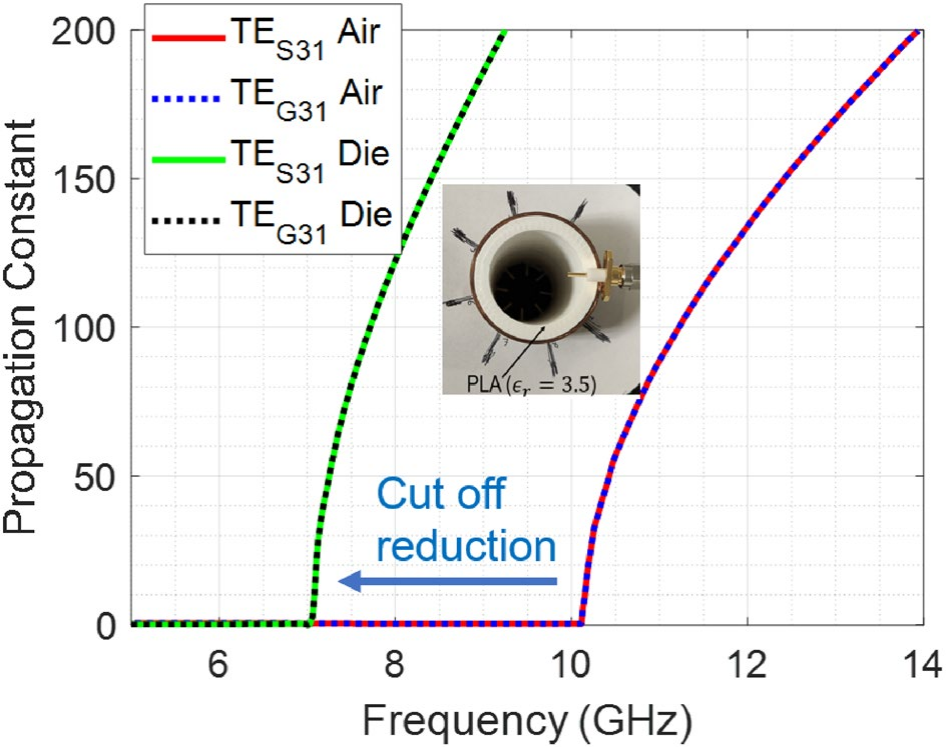
Dispersion relation of $\hbox {TE}_{31}$ mode with and without partial dielectric filling.

**Figure 9:**
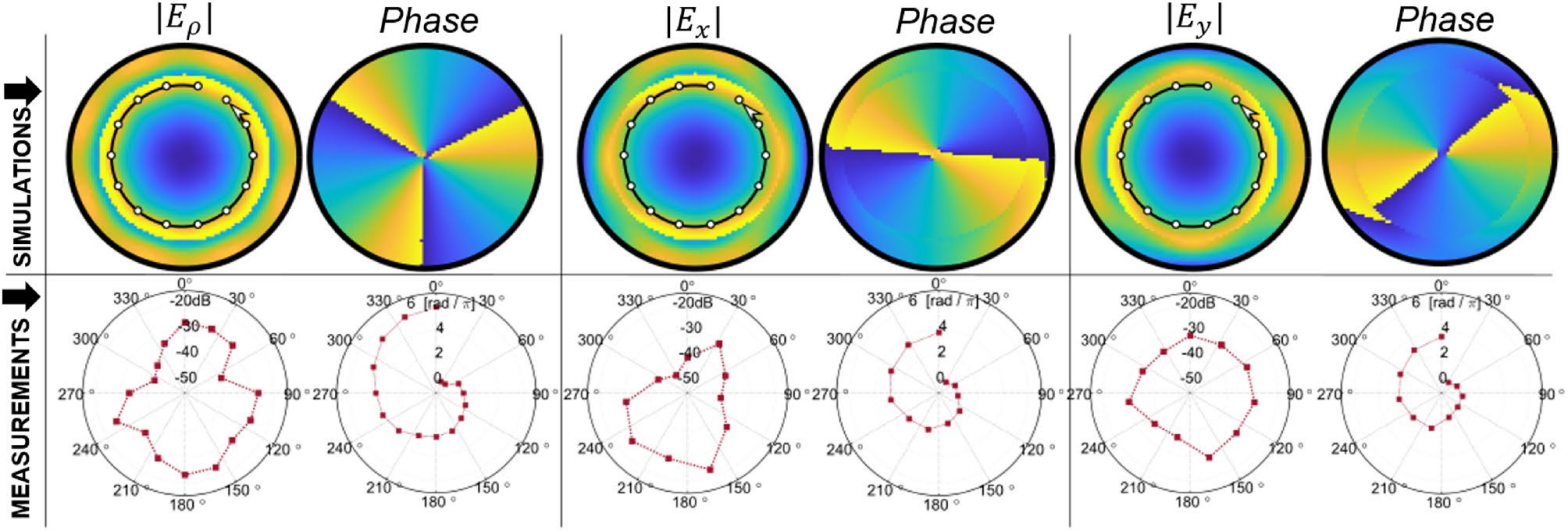
Numerical simulation confirming the synthesis of the vector vortex modes from degenerate TE modes in a perfectly electrical conductor (PEC) cylindrical waveguide. The excitation was based on wave-port excitation which allows perfect generation of TE modes in the cross-section. Vector vortex modes were generated by exciting two TE modes and introducing $90^o$ phase shift between the modes. Radius of the waveguide = 20 mm. Frequency=9.38 GHz.

### Measurement of VVM fields in partially dielectric filled waveguide

Demonstration of vector vortex modes in partially filled dielectric filled waveguide is shown in Fig. 9. This variation in the madia provides a method of manipulating the cut-off frequency of the waveguide and therefore allows design flexibility from a practical standpoint. Furthermore, this experimentation allows us to understand if non-metallic boundaries will cause disruption of electric and magnetic fields and if such disruption still allows generation of the VVW modes through the proposed antennas. We observe that due to presence of the dielectric filling, the cutoff frequency of the corresponding mode is decreased as shown in Fig. 8. Therefore, corresponding vector vortex modes can be obtained at a lower frequency as compared to a hollow waveguide of same outer perimeter. This is experimentally tested as shown in Fig. 9. The experimental set-up was similar to as shown in Fig. 6, but the prototype is partially filled with a dielectric. Dielectric insertion was of the form of a outer ring layer with 5 mm thickness 3D printed polylactic acid (PLA) material. Estimated dielectric constant of PLA is 3.5 with $tan(\delta )$ of 0.001^63,64^. The outer radius of the waveguide was chosen to be $a=$20 mm (shown in Fig. 8 inset).

Measurement results for this system and comparison with corresponding simulation plots is shown in Fig. 9. Simulation results showing electric field and phase at 9.38 GHz show that vector vortex modes were excited with a variation that a field discontinuity was observed at the dielectric-air interface for $E_x$ and $E_y$. Since $E_{\rho }$ components depending upon the tangential orientation of the field relative to the dielectric-air boundary. Since $E_{\rho }$ component is normal to the discontinuity, field profile is continuous in the entire cross section (Fig. 9. Spiral variation of phase along the azimuthal angle is observed from 0 to 6$\pi$ for $E_{\rho }$ component and from 0 to 4$\pi$ for $E_x$ and $E_y$ components. Reasoning for the phase variations for the three components holds true as was discussed above.

### Far-field measurement

**Figure 10:**
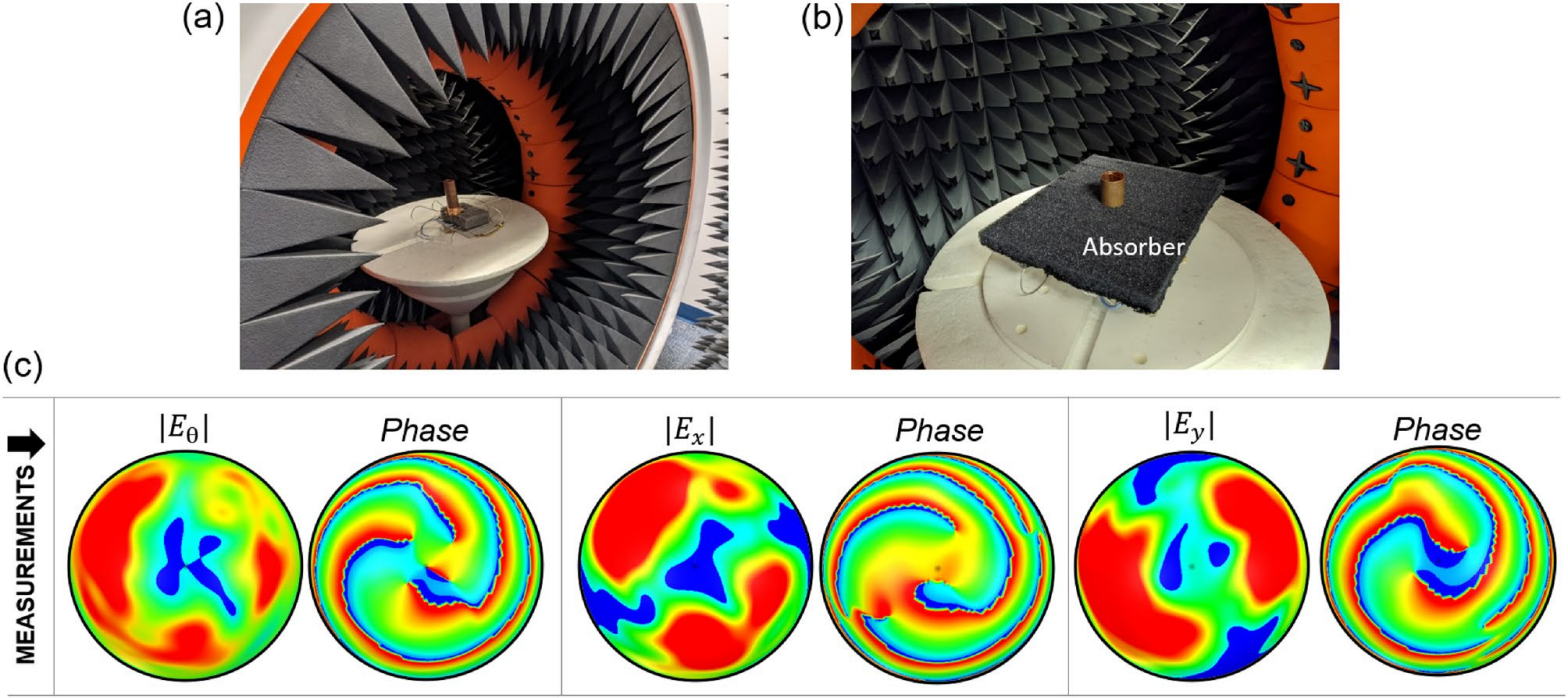
The vortex wave generating prototype inside Starlab Anechoic Chamber (**a**), wideband absorber to absorb the reflection from the cable and power divider (**b**) and the measured magnitude and phase values of $E_\theta$, $E_x$ and $E_y$ in spherical coordinate at 12 GHz (**c**).

This work further conducts measurement of the far-field radiation emanating from the cross-section of the waveguide. This particular measurement presents the utility of presented monopole-waveguide configuration towards wireless communication applications. We chose the hollow waveguide for the measurement setup and placed the prototype inside the Starlab Anechoic Chamber to see the field profile as shown in Fig. 10a. To avoid the unwanted reflections from the cables and the power divider, a wideband absorber is employed in such a way so that only the testing side of the waveguide is visible (Fig. 10b). Figure 10c shows the measured magnitude and phase of the measured $E_\theta$, $E_x$ and $E_y$ components of far-field. According to the discussion in the above sections, the vortex wave should have the central null in magnitude and phase values should vary from -$m\pi$ to +$m\pi$ for mode $\pm m$. The measured magnitude plot in Fig. 10c shows the central null on the magnitude plots and the spherical plots of phase-measurement confirm the generation of VVM mode m = +3 by showing three cycles of phase change from -$\pi$ to $\pi$, in total 6$\pi$ and left-handed spin on the $E_\theta$ phase plot. It is to be noted that $E_\theta$ is showed here in place of $E_\rho$. In the simulation setup, $E_\rho$ inside the waveguide represents vector moving away from origin in 2D plane (cross-section). But in far-field, $E_\rho$ is normal to surface of measurement and antenna far-field has zero $E_\rho$ component. So, addition of $E_\theta$ is considered which in this context is a close representation of $E_\rho$ (of 2D planar coordinate) fields in the far-field 3D coordinate. For $E_x$ and $E_y$ components, 0 to 4$\pi$ azimuthal angle variations are observed which confirms the generation of $m=-3$ mode. Little distortion on the magnitude and phase plots are introduced due to the fabrication and measurement tolerance. Moreover, the prototype is designed to generate the vorticity inside the waveguide, so little distortion is expected in the farfield results.

## Discussion

We demonstrated the use of radially aligned antenna elements inside cylindrical waveguides to produce vector vortex modes. The paper demonstrated via numerical and experimental means that the vector vortex modes can be excited and propagated in a cylindrical waveguide through a proper selection of antenna locations and excitation phases within the waveguides. Analysis of dispersion relation shows that the cutoff frequencies for these modes follow the cut-off frequencies of the underlying TE modes, and this applies to the proposed system of antenna elements as well. Furthermore, it was demonstrated that such vector vortex modes also exist in partially dielectric-filled waveguides, such that the cut-off frequency of the modes is reduced due to effective increase in the electrical size of the waveguide. Above observation is then validated through experimental demonstrations. Proposed system of antenna elements, guidance of phase excitation, and the proposed formulation given for minimum number of antenna elements needed, would serve as a guidelines for future applications where generation and propagation of vector vortex modes in cylindrical waveguides is desired.

## Data Availability

All data generated or analysed during this study are included in this published article. The raw data used and/or analysed during the current study available from the corresponding author on reasonable request.
